# Interactions between β-arrestin proteins and the cytoskeletal system, and their relevance to neurodegenerative disorders

**DOI:** 10.3389/fendo.2023.957981

**Published:** 2023-02-09

**Authors:** Tibor Szénási, Gábor Turu, László Hunyady

**Affiliations:** ^1^Institute of Enzymology, Research Center for Natural Sciences, Centre of Excellence of the Hungarian Academy of Sciences, Budapest, Hungary; ^2^Department of Physiology, Faculty of Medicine, Semmelweis University, Budapest, Hungary

**Keywords:** arrestin, microtubule, Alzheimer’s disease, actin, cytoskeleton, neurodegeneration

## Abstract

β-arrestins, which have multiple cellular functions, were initially described as proteins that desensitize rhodopsin and other G protein-coupled receptors. The cytoskeletal system plays a role in various cellular processes, including intracellular transport, cell division, organization of organelles, and cell cycle. The interactome of β-arrestins includes the major proteins of the three main cytoskeletal systems: tubulins for microtubules, actins for the actin filaments, and vimentin for intermediate filaments. β-arrestins bind to microtubules and regulate their activity by recruiting signaling proteins and interacting with assembly proteins that regulate the actin cytoskeleton and the intermediate filaments. Altered regulation of the cytoskeletal system plays an essential role in the development of Alzheimer’s, Parkinson’s and other neurodegenerative diseases. Thus, β-arrestins, which interact with the cytoskeleton, were implicated in the pathogenesis progression of these diseases and are potential targets for the treatment of neurodegenerative disorders in the future.

## 1 Introduction

The diverse functions of the cytoskeletal system include cell protection, cytokinesis, cell motility, intracellular transport, cell division, and organization of organelles within the cell. In eukaryotes, three types of cytoskeleton exist: microtubules, actin microfilaments, and intermediate filaments. The clinical importance of these systems is indicated by the findings that their dysfunction may initiate neurodegenerative or cancerous processes (1).

Arrestins were first discovered as proteins responsible for quenching the light-induced photoreceptor response in the retina (2). Activated rhodopsin is phosphorylated by rhodopsin-kinase on serine/threonine (S/T) amino acids at its C-terminus. This visual arrestin recognizes and binds to the phosphorylated rhodopsin and uncouples it from its heterotrimeric G-protein partner, transducin. Later, it was discovered that β2 adrenergic receptor (β2AR) shares the seven-transmembrane topology with the rhodopsin protein. It has also been recognized that β2AR undergoes similar phosphorylation as rhodopsin, and the kinase responsible for this modification was called β2-adrenergic receptor kinase (βARK1), which is now named G protein-coupled receptor kinase 2 (GRK2). Two different visual receptor kinases (GRKs) and arrestins (arrestin1 and 4) were identified, together with five additional non-visual GRKs and two arrestins (arrestin2 and 3, also called β-arrestin1 and β-arrestin2) (3). Non-visual arrestins are expressed in all tissues. They bind to activated and phosphorylated GPCRs and uncouple them from their cognate heterotrimeric G-proteins. In the case of many GPCRs, this process is followed by β-arrestin-mediated transportation to the clathrin-coated pits and internalization of the receptor (4). β-arrestins are localized to the cytoplasm and, in the case of the β-arrestin1, also to the nucleus, which localization is quickly changed upon stimulation. First, β-arrestins translocate to the cell membrane and bind to the receptors, and then, they follow the receptors to clathrin-coated pits or even to intracellular vesicles (see below). β-arrestins form two interactions with GPCRs: they can bind to the phosphorylated C-terminal part of the receptors, and/or their finger loop domain is inserted into the transducer-binding region of the receptor, forming the so-called “core-interaction” (3, 5–8).

Based on the strength of the binding between β-arrestins and GPCRs, two groups of receptors can be distinguished: class A receptors bind to β-arrestins with weaker affinity, and the interaction is restricted to the vicinity of the cell membrane, while class B receptors bind β-arrestins with high affinity and remain bound to them even in the endosomes (9). The interaction stability is determined by the C-terminal interaction, exchanging the C-termini between receptors can switch class A receptors to class B types, and vice versa (9). The interaction stability is determined by interactions between positively charged amino acids in the β-arrestin and the phosphorylated serine-threonine amino acids on the receptor C-terminus, or in the case of some receptors, on the third intracellular loop (10–15). β-arrestins are critical regulators of multiple cellular processes and signaling pathways, such as scaffolding, receptor desensitization, GPCR endocytosis and trafficking, transcriptional regulation, cell growth and survival, cytoskeletal rearrangement and cell migration, and other specialized functions (16–19). β-arrestins exist in distinct conformational states: free, receptor-bound, microtubule-associated (20), and associated with other partner proteins. The interactome of β-arrestin proteins includes the prominent component proteins for the three major type elements of the cytoskeleton: actins for the actin filaments, tubulins for the microtubules, and vimentin for the intermediate filaments (16). These cytoskeleton-associated proteins, including multiple isoforms of actins, are abundant in cells, and increasing evidence indicates that the interaction between these proteins and β-arrestins may have functional consequences.

## 2 Interactions of β-arrestin proteins and the cytoskeleton

### 2.1 Interactions between β-arrestin proteins and the microtubular system

**Figure 1:**
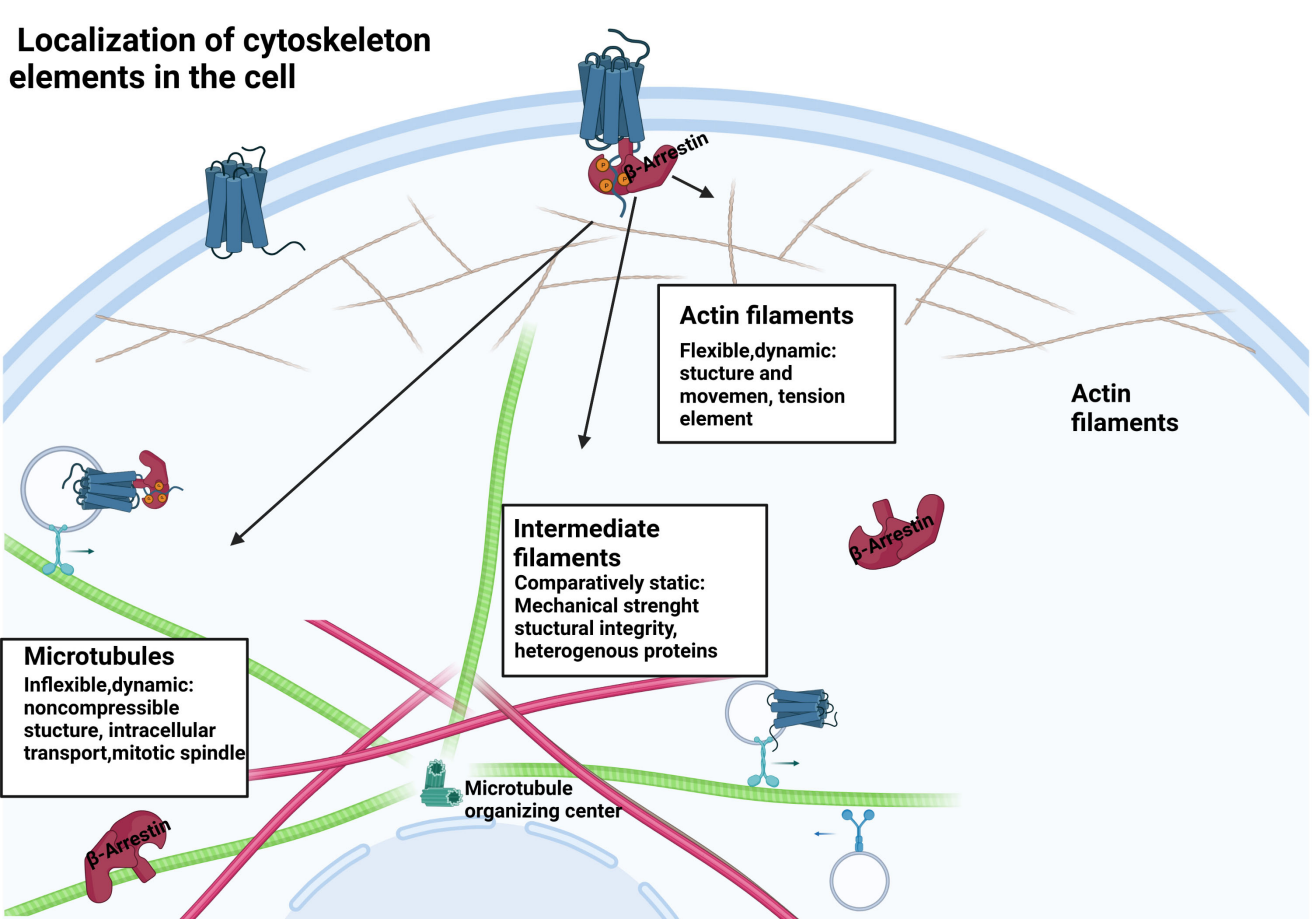
β-arrestin protein location within the cell. β-arrestins exist in distinct conformational states: free, receptor-bound, and microtubule-associated.

**Figure 2:**
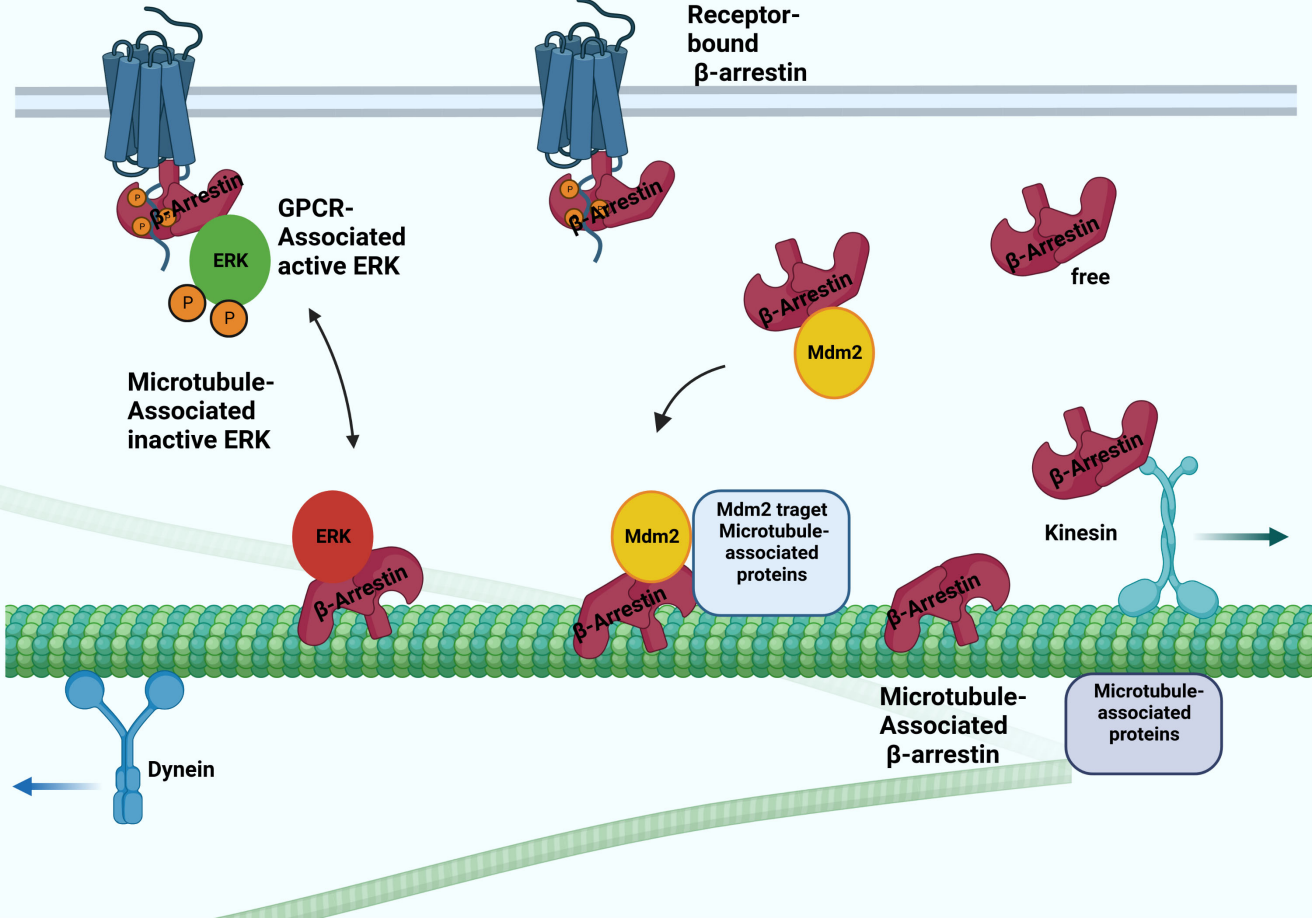
Possible interactions between arrestins and microtubular system. The β-arrestin proteins binding to microtubules involve the conformation change. They bind to the microtubule system and regulate their activity by recruiting ERK and Mdm2. The β-arrestin2 interacts with the kinesin-like protein KIF3A motor protein.

The microtubule network, an integral component of the eukaryotic cytoskeleton, plays a critical role in cell division, differentiation, maintenance of cell shape and polarization, motility and intracellular transport, and formation of pathological inclusion bodies (21, 22) (**Figures 1**, **2**). Microtubules (MT) are composed of the highly conserved dimers of α- and β-tubulin and form the microtubule network (22). Dynamic instability is characteristic of microtubules and is central to their functions by allowing their rapid spatial and temporal reorganization and differentiation in response to environmental factors or signals (23).

Interaction with the microtubular system was first demonstrated *in vivo* in the case of arrestin1, a visual arrestin, in rod photoreceptors, and it has been reported that it determines the differential subcellular localization of its splice variants (24). The interaction of rod arrestin with the microtubule system is increased in the dark. The light-dependent but energy-independent translocation of rod arrestin is involved in light and dark adaptation (25). Interestingly, visual arrestin N- and C-domains or truncated arrestin(1-378) bind microtubules substantially better than the full-length protein. The MT-bound arrestin’s conformation is different from the free and receptor-bound conformations. The exchange of two elements on the concave sides of the N- and C- terminal domains reverses both the receptor specificity and the relative ability of rod-arrestin and β-arrestin1 to bind microtubules, indicating that the receptor- and MT-binding sites overlap (26). Thus, β-arrestin proteins cannot interact with microtubules and receptors simultaneously. It was also shown that MT-bound arrestin recruits extracellular signal-regulated kinase 2 (ERK)1/2 proteins and mouse double minute 2 (MDM2), an E3 ubiquitin ligase, and regulates their activity: the translocation ERK to MTs by β-arrestin1 reduces the level of ERK activation (27). Although β-arrestin1 and rod-arrestin bind both polymerized and unpolymerized tubulin, they do not affect microtubule polymerization. These reports described a new non-receptor binding partner of arrestin proteins and showed that the interaction between arrestins and the microtubule system localizes specific signaling molecules to the cytoskeleton. β-arrestin-dependent recruitment of the protein kinase ERK silences ERK, whereas its recruitment to the ubiquitin E3 ligase Mdm2 directs Mdm2 to its cytoskeletal substrates (**Figure 2**). α-methylserotonin, an agonist of 5HT2A/C receptors, caused a β-arrestin-dependent stimulation of ERK1/2 phosphorylation in the dendrites of cortical neurons. The siRNA-mediated depletion of one of the β-arrestins suppressed this process. The depletion also counteracted the serotonin 5HT-1A receptor-mediated disruption of microtubule stability, which is required for normal N-methyl-D-aspartate (NMDA) receptor transport (28). On the other hand, targeting Mdm2 to microtubules by β-arrestin redirects Mdm2 activity toward MT-associated proteins, significantly increasing partner ubiquitination (27).

**Figure 3:**
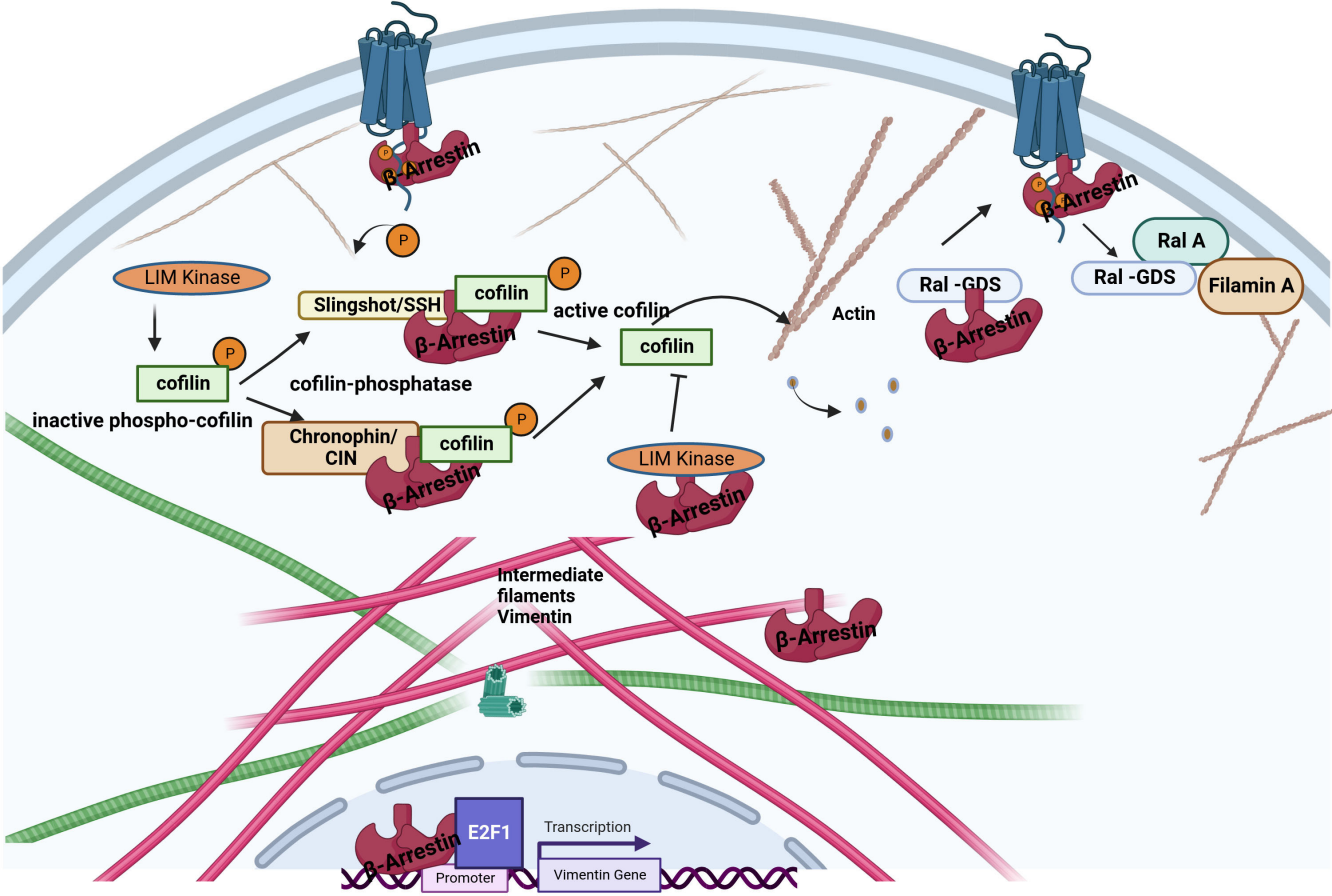
Interaction between β-arrestin proteins and other cytoskeletal elements. β-arrestins directly bind actin filaments and vimentin. Increased cofilin activity results from the scaffolding of cofilin by the upstream cofilin phosphatases chronophin and slingshot. The increased cofilin activity is also related to LIMK inhibition by arrestins. Older filaments are cut by cofilin, producing new filament seeds. β-arrestin2 binds to the guanine exchange factor for GTPase, Ral1(Ral-GDS). After receptor activation, RAL-GDS is released, which activates Ras-related protein Ral-A (RalA). RalA interacts with Filamin-A to promote actin rearrangement. β-arrestin1 can enhance vimentin gene expression in association with the transcription factor E2F1.

It has been first reported that rod arrestin can form oligomers at physiological concentrations, and monomeric visual arrestin binds to rhodopsin, whereas microtubules can bind both arrestin1 monomers and tetramers (29). β-arrestin1 exhibited dimerization throughout crystallization (30), indicating that it may oligomerize (31). Oligomerization of β-arrestin1 is facilitated in the presence of inositol-hexakisphosphate (IP6) (32, 33). The purpose of β-arrestin1 oligomerization remains poorly understood. Besides thecytoplasmic and plasma membrane (in activated cells) localization of β-arrestin1, its presence in the nucleus was also reported (34). β-arrestin2 does not localize to the nucleus since the C-terminus of β-arrestin2, in contrast to that of β-arrestin1, has a nuclear export signal (35, 36). However, hetero-oligomerization with β-arrestin2 dramatically changed the subcellular distribution of β-arrestin1, which became predominantly cytosolic (37). It has also been reported that although monomeric β-arrestin1 can enter the nucleus, its homo-oligomers are mostly cytosolic (32), suggesting that homo- and hetero-oligomerization of β-arrestin1 cause similar changes in its subcellular localization. A proteomics-based screening study on the arrestin interactome revealed that over three hundred distinct proteins coprecipitated with β-arrestin1 or β-arrestin2 under different conditions (16). This study confirmed the interaction of β-arrestin proteins with tubulin and has identified new interactions with resident and accessory cytoskeletal proteins. Interestingly, β-arrestin1 binds kinesin family member 3A (KIF3A) and filamin-A, whereas β-arrestin2 binds cofilin-1 (actin assembly protein) (16) (**Figures 2**, **3**) and localizes in primary cilia (38, 39). Primary cilia are antenna-like structures on the surface of most vertebrate cells, which regulate essential signaling pathways during development and play crucial roles in tissue homeostasis (40). Lack of β-arrestin2 resulted in ciliogenesis defects and uncontrolled proliferation in β-arrestin2 KO mouse embryonic fibroblast (MEF) cells (39). β-arrestin2 interacts with 14-3-3 proteins and kinesin KIF3A (39), and β-arrestin proteins are required for the formation of a “translocation complex”, including KIF3A and Smo, that promote activation of downstream transcriptional targets in mammalian cells and activity-dependent localization of Smo to primary cilia (38). Microtubule-independent localization of β-arrestin2 to the proximal region of centrioles has been reported (38). Endogenous β-arrestin2 is localized at the centrosome during the entire cell cycle, and β-arrestin2 is co-localized with the γ-tubulin and acetyl-tubulin staining (41).

### 2.2 β-arrestin interactions with actin

The actin cytoskeleton is responsible for mediating various cellular processes, including cell structure determination, organelle transport, cell migration, axonal growth, and endocytosis or phagocytosis. Reorganization of the cytoskeleton is a crucial process during alterations of cell shape, signal transduction, movement, and many other dynamic cellular processes. Mitogen-activated protein (MAP) kinase, c-Jun N-terminal kinase, and p38 kinases play critical roles in regulating dynamic changes of the cytoskeleton because several extracellular signals that promote cell shape change converge on MAP kinases, which phosphorylate downstream targets involved in the regulation of the actin cytoskeleton (42). During cell migration, the actin filaments are rapidly reorganized by the formation of new barbed ends and by cutting and severing existing filaments. This reorganization occurs partly through the binding of various actin cross-linking and assembly proteins (43). Bhattacharya et al. have identified by co-immunoprecipitation and yeast two-hybrid screening that Ral-GDS, a stimulator of Ral-GDP dissociation, is a β-arrestin-binding protein in human neutrophil leukocytes (44). These authors proved that chemoattractant receptor-induced regulation of β-arrestin-Ral-GDS protein complexes is necessary for Ral activation, leading to cytoskeleton reorganization. After the recruitment of β-arrestins to the activated GPCR, Ral-GDS dissociates from the β-arrestin and becomes activated (45). Ral-GDS stimulates RalA GTPase at the leading edge of the actin cytoskeleton where the β-arrestin-ERK scaffolds are located. After GPCR activation, β-arrestins also induce stress fiber formation and reorganization of the actin cytoskeleton by interacting with RhoGEFs or RhoGAPs (46). Stress fiber formation and actin structure polymerization are required for cell movement, adhesion, and chemotaxis (47). After disruption of the actin cytoskeleton dynamics by toxins β-arrestin2, but not β-arrestin1, could rescue agonist-induced endocytosis of the β isoform of the thromboxane A2 receptor (TPβ). The use of β-arrestin2 mutants showed that this was an AP2- and a clathrin-dependent mechanism (48).

Double β-arrestin1 and 2 double knockout MEFs have been used to study β-arrestin function for over two decades (49). These cells have an unusual size and shape and are dramatically different from wild-type MEF cells. The actin cytoskeletal system of β-arrestin KO MEF cells, plated on fibronectin, is also different from that of the wild-type cells, and the size of these cells is much larger. However, retroviral expression of β-arrestin1 or 2 rescued the morphological phenotype of the double β-arrestin1 and 2 KO MEF cells (50).

### 2.3 β-arrestin interactions with intermediate filaments

The intermediate filaments include vimentin, a structural protein found in mesenchymal cells (51). A vimentin monomer has non-helical N-terminal and C-terminal domains and a central α-helical domain (52). The interaction between β-arrestins and vimentin was first reported by Xiao et al. based on co-immunoprecipitation and mass spectroscopy data (16). Pillai et al. have demonstrated that β-arrestin1, but not β-arrestin2, is required for nicotine-induced epithelial-mesenchymal transition and metastasis formation. The increased levels of β-arrestin1, vimentin, and fibronectin in tumors from smokers and nonsmokers suggest that these molecules contribute to the growth and progression of non–small cell lung cancer cells. At the same time, the induction of these genes may be an essential mechanism by which nicotine exerts its tumor-promoting functions. Interestingly, β-arrestin1 was required to induce mesenchymal promoters following stimulation by the nicotinic acetylcholine receptor but played no role in the TGFβ-mediated induction of the vimentin and fibronectin genes. The β-arrestin1 may be co-operated with the E2F1 transcription factor in the transcriptional regulation of these genes (53).

### 2.4 Roles of β-arrestin proteins in the regulation of cell migration and chemotaxis

Filamin-A, an actin cross-linking protein, can serve as a scaffold for several binding partners and is a crucial regulator of cell migration. Similar to β-arrestins, this protein can localize various kinases at the leading edge and interacts with β-arrestins downstream of the angiotensin receptor to control chemotaxis, indicating that the interaction between these two proteins may reach different levels of actin dynamics during cancer cell motility (54, 55). Downregulation of β-arrestin causes decreased GPCR internalization and chemotaxis (56).

Cofilin is one of the essential proteins which participate in the dynamic reorganization of the cytoskeleton (57). The binding of cofilin to actin filaments causes its cleavage, and the number of ends increases, either shortening or lengthening the filament, depending on the physiological situation. Cofilin is a target of the opposing actions of LIM kinase, which inactivate it by phosphorylation at Ser3, and specific phosphatases, such as slingshot (SSH) and chronophine (CIN), which activate it, enabling the fine-tuning of the regulation of the actin cytoskeleton dynamics (**Figure 3**) (57, 58). β-arrestins are tight regulators of cofilin phosphorylation in response to upstream GPCR stimuli by interacting with LIM kinase and Ser/Thr phosphatases, including slingshot and chronophine, to control the direction and force of membrane expansion required for cell movement (19, 59). The degree of control depends on the GPCR and the cellular context. During chemotaxis, chemotactic signals, such as those from PAR-2 stimulation, promote the local formation of free actin barbed ends, membrane protrusions, and leading-edge formation by interacting with cofilin and phosphatases or directly binding to LIM kinase to antagonize its ability to phosphorylate and inactivate cofilin (54, 60). β-arrestin2 has also been shown to negatively regulate the migration of dendritic cells and play a role in inflammatory diseases (61).

## 3 The role of β-arrestin proteins in cytoskeleton-associated neurodegenerative disease

Neurodegenerative diseases cause progressive neuronal dysfunction, toxicities, and death (62). The most common of these are Alzheimer’s disease (AD), Parkinson’s disease (PD), frontotemporal dementia (FTD), and Huntington’s disease (HD). GPCRs and cytoskeletal proteins play important roles in these disorders and can also serve as therapeutic targets (1, 62). Abnormal aggregates of cytoskeletal proteins are pathologic signatures of neurodegenerative diseases. These aggregated proteins of the neuronal cytoskeleton characteristically include aggregates of the neuronal intermediate filaments and inclusions containing the microtubule-associated protein (MAP) tau protein (1). The pathological features of AD have three main parts, 1) extracellular senile plaques due to the deposition of beta-amyloid peptide (Aβ), 2) intracellular neurofibrillary tangles due to tau phosphorylation, and 3) neuronal cell death (63, 64). Aβ, a 4 kDa fragment of the amyloid precursor protein (APP), is widely produced by brain neurons and astrocytes to a lesser extent. Two sequential proteolytic cleavages of APP by β-secretase at the ectodomain and γ-secretase at the intramembrane site generate Aβ (65). MAP proteins, such as tau, regulate the extent and rate of microtubule assembly and play important roles in morphogenetic processes, such as axonal outgrowth (66). In AD, tau is phosphorylated at sites that are normally not phosphorylated in adulthood or is significantly phosphorylated at sites that are normally phosphorylated in adult tau (67). Arrestin proteins regulate these and other proteins implicated in the development of neurodegenerative diseases.

Changes in β-arrestin and GRK expression were first reported in Parkinson’s disease with dementia (68). Amyloid-β peptide (Aβ) can bind to the β2-adrenergic receptor and cause allosteric receptor activation leading to cAMP- and arrestin-mediated signaling (69, 70). Aβ treatment can activate the β2-adrenergic receptor-arrestin-MAPK pathway in prefrontal cortex primer neurons. It was reported that MEK, an upstream regulator of ERK1/2 MAP kinases, promotes the phosphorylation of tau, and β-arrestin2 is required for the Aβ-induced ERK1/2 phosphorylation (71). These data suggest that Aβ-induced MEK phosphorylation simultaneously leads to β-arrestin2-dependent ERK1/2 activation and arrestin-independent tau phosphorylation (71).

**Figure 4:**
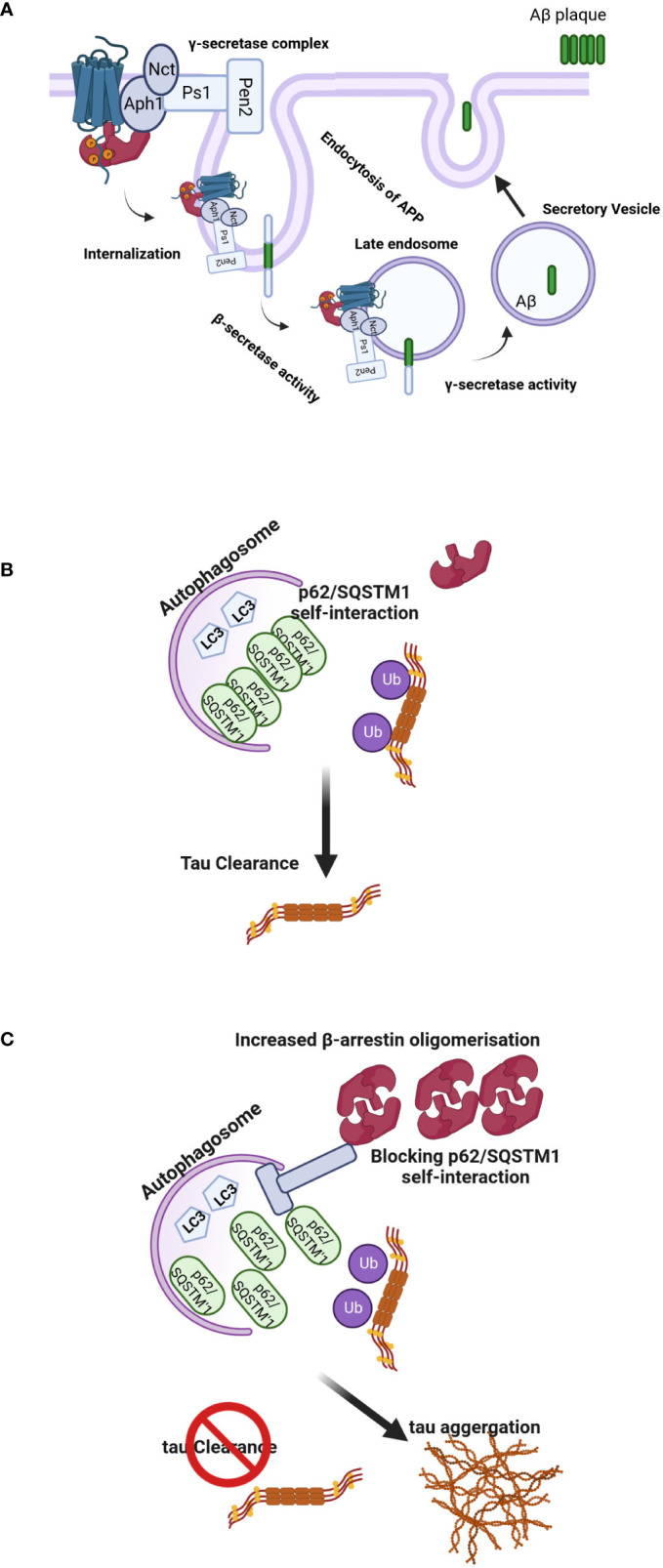
**(A)** Regulation of Aβ-related pathology by β-arrestin2. Activation of GPCRs by their ligands increases the affinity for β-arrestin2. Recruitment of β-arrestin2 to GPCRs ends up interacting with the γ-secretase complex *via* the Aph1 subunit. β-arrestin2 mediates the internalization of GPCRs and localizes γ-secretase to late endosomes, where the acidic environment often increases its activation. APP is cleaved by the β-secretase, and C-terminal APP fragments are produced, the direct substrates of the γ-secretase. These fragments are often subsequently cleaved by the γ-secretase to provide Aβ and APP intracellular domains. The produced Aβ is often discharged into intercellular space *via* secretory vesicles, resulting in extracellular amyloid plaque formation. **(B, C)** Model of β-arrestins-promoted tauopathy in AD and FTD. In healthy brains, monomeric β-arrestins regulate GPCR trafficking, and there is no excess of oligomeric β-arrestins, and thus misfolded tau is efficiently ubiquitylated and targeted for autophagy clearance. However, in FTD form-tau brains, β-arrestins oligomers are increased, inhibiting p62/SQSTM1-mediated autophagy, leading to failure of misfolded/aggregated tau to be efficiently cleared.

The γ-secretase complex plays a pivotal role in the production of the Aβ. Undertanding the processes involved in its regulation might be essential to developing new therapies (72). The γ-secretase complex is composed of four integral membrane proteins: the catalytic component presenilin 1(Ps1) or 2 (Ps2) and the essential cofactors nicastrin (Nct), anterior pharynx defective 1 (Aph1) and presenilin enhancer 2 (Pen 2) (73). It has been proposed that β-arrestin1 plays a role in AD pathogenesis through the regulation of this complex since depletion of β-arrestin1 expression by specific siRNA reduced γ-secretase activity and Aβ40 and Aβ42 production in neurons. Moreover, when APP/PS1 mice, a model of the AD (74), were crossed with β-arrestins1−/− mice, offspring had lower amounts of Aβ deposits compared to β-arrestins1+/+ mice and ameliorated memory deficits (75). In other experiments, Thathiah et al. isolated embryonic neuronal cultures from wild-type and β-arrestin proteins knock-out mice. The amounts of endogenous Aβ40 and Aβ42 were substantially reduced in β-arrestins2−/− but not β-arrestins1−/− neurons, suggesting that β-arrestin2 is involved endogenously in the modulation of neuronal Aβ production (76). Although the role of the β-arrestin1 in these two experiments is somewhat controversial, the different timescale of the siRNA vs. knockout models might explain these differences. Nevertheless, the levels of both β-arrestin proteins were increased in the brains of patients with AD (75, 77) (**Figure 4A**). Pontrello et al. have demonstrated that deletion of β-arrestin2 can protect against Aβ-induced loss of dendritic spines in hippocampal neurons, further supporting the role of the β-arrestin2 in the pathogenesis of AD (78). Overexpression of both β-arrestins can cause an increase in Aβ peptide generation (75, 76). β-arrestins interact with the Aph1a subunit of the γ-secretase complex but not with Nct, Ps1, and Pen2 subunits offering a mechanism through which the effects of the arrestin could be explained (75, 76).

The other mechanism by which β-arrestins might regulate the amount of Aβ and tau is the degradation of these proteins, further complicating the picture. Both Aβ and tau proteins can be degraded by the ubiquitin-proteasome system (UPS) and autophagy-lysosome system (ALS), a process called clearance of Aβ and tau (64). The upregulation of β-arrestin1 and β-arrestin2 was an early event after Aβ25-35 exposure, which was accompanied by induction of autophagy. Downregulation of β-arrestin1 resulted in decreased autophagic flux and enhancement of Aβ25-35 induced cell death, whereas depletion of β-arrestin2 reversed to some extent Aβ25-35 cytotoxicity. Liu et al. demonstrated that β-arrestin2, but not β-arrestin1, was critical for autophagy activation and preferentially regulated the nicotinic acetylcholine receptor α7 subunit expression in the membrane, which is responsible for the neuroprotective effect of nicotine. Suppression of β-arrestin2 increased the plasma membrane expression of this receptor, attenuating Aβ25-35 toxicity (79).

Interestingly, oligomerization of β-arrestin2 is required for tau stability. Overexpression of β-arrestin2 in tau-expressing HeLa cells inhibited the bafilomycin A-induced increase in LC3-positive puncta, suggesting that β-arrestin2 inhibits autophagy at or before LC3. The β-arrestin2 oligomers increase tau levels by blocking the self-interaction of p62, which is the initial step essential for autophagy flux. These data have demonstrated that the oligomerized form of β-arrestin2 reduces the elimination of tau protein by interfering with p62-mediated autophagy (**Figures 4B, C**). The p62/SQSTM1 interacts with polyubiquitinated tau through its ubiquitin-associated domain and serves a unique role in the regulation of tau proteasomal degradation (80). Thathiah et al. have proposed that the development of small molecule β-arrestin2 oligomerization inhibitors may have therapeutic relevance for intervention in frontotemporal dementia (FTD) form-tau to enhance the elimination of the tau protein without exerting significant side effects *via* GPCR signaling pathways. Agents that promote the degradation of misfolded aggregated proteins, such as tau, are attractive therapeutic targets for neurodegenerative diseases (81). Cargo-bound p62/SQSTM1 promotes autophagosome maturation by converting LC3 to its lipidated active form, LC3.II (82). This data is in agreement with the inhibitory effect of β-arrestin1 on the formation of LC3 puncta and the reduction in p62-LC3 colocalization. These data are consistent with the observed role of β-arrestin1 in impeding p62/SQSTM1 flux and impairing destruction of misfolded tau (83) and underline the potential of β-arrestin proteins as drug targets for the therapy of neurodegenerative diseases. FTD includes a spectrum of clinical syndromes associated with various neurodegenerative diseases. In patients with FTD, the primarily affected regions are the frontal and temporal lobes (84). Therefore, FTD is also regarded as frontotemporal lobar degeneration. β-arrestin2 protein and mRNA were significantly increased in the tau-FTD brain samples compared to the controls and in the brain samples of the P301S transgenic mice compared to the brains of the non-transgenic mice (85). A recent report has shown that β-arrestin1 levels are increased in the brains of FTD patients, and β-arrestins are essential for the β2 adrenergic receptor and mGluR2 glutamate receptor-mediated increase in pathogenic tau (83). Increased β-arrestin1 also causes the accumulation of pathogenic tau, whereas its reduction alleviates tau-induced pathology and rescues the impaired synaptic plasticity and cognitive abilities in PS19 mice. Biochemical and cellular studies show that these effects of β-arrestin1 were mediated by the destabilization of microtubules and impairment of p62/SQSTM1 autophagy flux due to the interference with p62/SQSTM1 body formation, which promotes pathogenic tau accumulation.

Other β-arrestin partner proteins have been also implicated in the pathogenesis of neurodegenerative diseases, such as ERK, Filamin-A, and cofilin (86, 87). For example, the activated form of cofilin, a partner of both β-arrestin proteins, exacerbates tau pathology by interfering with tau-mediated microtubule dynamics (88) and may contribute to the cytoskeletal pathogenesis in Alzheimer’s disease (87).

In Parkinson’s disease, β-arrestin proteins play a role in the microglia-mediated inflammation and the pathogenesis of this disease (89). The E3 ubiquitin protein ligase Parkin directly interacts with both β-arrestins, and Parkin promotes Mdm2 binding to β-arrestin proteins (90). Interestingly, certain anti-Parkinson’s disease drugs (e.g. levodopa and piribedil) induced β-arrestin2 and dopamine D2 receptor-mediated Aβ elevation in Alzheimer’s disease model cells (91). On the other hand, in macaque and mouse models of Parkinson’s disease, β-arrestin2 overexpression has been shown to ameliorate dyskinesia symptoms, which develop in patients during levodopa treatment (92).

## 4 Concluding remarks and future perspectives

The interactions between β-arrestin proteins and the cytoskeleton, and its assembly proteins that determine the dynamics of the skeleton, are critical mechanisms for several physiological and pathological processes. The β-arrestinome (93), the interactome of β-arrestin proteins, contains more than 400 cytosolic and nuclear protein partners, suggesting various functions for these proteins. Interactions between β-arrestin proteins and the cytoskeletal system play essential roles in neurodegenerative diseases, especially Alzheimer’s. In addition, due to their role in cytoskeletal rearrangement and movement, β-arrestin proteins may also be relevant targets for cancer therapy. Experimental approaches to inhibit arrestin function by cell-specific genetic ablation or downregulation by siRNA have been pursued, including aptamers, the oligonucleotides with structures that allow selective binding to the surface of pathological target proteins and inhibit protein-protein interactions. Arrestin2-specific nucleotide aptamers have been developed, and Kotula et al. have reported that β-arrestin2-specific aptamers not only interfere with β-arrestin-dependent signaling but also inhibit the malignant progression in leukemic cell models and samples from human patients (94). Therefore, there is a need for novel approaches that exploit the therapeutic potential of β-arrestin2 without interfering with their physiological roles. Instead of small molecules, targeting β-arrestin oligomers or cell- specific targeting of these proteins may lead to novel therapeutic approaches to treat neurodegenerative and other diseases.

## Author contributions

All listed authors have made a substantial, direct, and intellectual contribution to the work and have approved it for publication.
